# Racial Differences in Incident Genitourinary Cancer Cases Captured in the National Cancer Database

**DOI:** 10.3390/medicina57070671

**Published:** 2021-06-29

**Authors:** Dylan T. Wolff, Thomas F. Monaghan, Danielle J. Gordon, Kyle P. Michelson, Tashzna Jones, Raymond Khargi, Matthew T. Smith, Fenizia Maffucci, Hyezo Kwun, Nicholas R. Suss, Andrew G. Winer

**Affiliations:** 1 Department of Urology, Wake Forest School of Medicine, Winston-Salem, NC 27101, USA; dylantwolff@gmail.com; 2 Department of Urology, University of Texas Southwestern Medical Center, Dallas, TX 75390, USA; 3 Department of Urology, SUNY Downstate Health Sciences University, Brooklyn, NY 11203, USA; danielle.gordon@downstate.edu (D.J.G.); raymond.khargi@downstate.edu (R.K.); matthewsmith445@gmail.com (M.T.S.); andrew.winer@downstate.edu (A.G.W.); 4 Department of Urology, Kings County Hospital Center, Brooklyn, NY 11203, USA; 5 Department of Urology, University of South Florida, Tampa, FL 33606, USA; kyle.p.michelson@gmail.com; 6 Department of Urology, Yale University School of Medicine, New Haven, CT 06520, USA; tashzna.jones@yale.edu; 7 Department of Urology, Temple University Hospital, Philadelphia, PA 19104, USA; fenizia.maffucci@gmail.com; 8 Division of Urology, Perelman School of Medicine at the University of Pennsylvania, Philadelphia, PA 19104, USA; hyezo.kwun@pennmedicine.upenn.edu; 9 Department of Surgery, University of Chicago, Chicago, IL 60637, USA; nicholasr.suss@gmail.com

**Keywords:** access, bladder, disparity, kidney, NCDB, penile, prostate, race, testicular, USCS

## Abstract

*Background and Objectives*: The National Cancer Database (NCDB) captures nearly 70% of all new cancer diagnoses in the United States, but there exists significant variation in this capture rate based on primary tumor location and other patient demographic factors. Prostate cancer has the lowest coverage rate of all major cancers, and other genitourinary malignancies likewise fall below the average NCDB case coverage rate. We aimed to explore NCDB coverage rates for patients with genitourinary cancers as a function of race. *Materials and Methods*: We compared the incidence of cancer cases in the NCDB with contemporary United States Cancer Statistics data. *Results*: Across all malignancies, American Indian/Alaskan Natives subjects demonstrated the lowest capture rates, and Asian/Pacific Islander subjects exhibited the second-lowest capture rates. Between White and Black subjects, capture rates were significantly higher for White subjects overall and for prostate cancer and kidney cancer in White males, but significantly higher for bladder cancer in Black versus White females. No significant differences were observed in coverage rates for kidney cancer in females, bladder cancer in males, penile cancer, or testicular cancer in White versus Black patients. *Conclusions*: Differential access to Commission on Cancer-accredited treatment facilities for racial minorities with genitourinary cancer constitutes a unique avenue for health equity research.

## 1. Introduction

The National Cancer Database (NCDB) is a longstanding joint initiative of the American College of Surgeons Commission on Cancer (CoC) and the American Cancer Society that provides a wealth of information on cancer diagnosis, treatment, and outcomes [1,2].

Investigative interest in the NCDB for addressing pressing knowledge gaps in oncology has grown exponentially in recent years [3]. As one of the largest clinical cancer registries in the world, the NCDB is indeed a powerful tool for oncology research, capturing nearly 70% of all new cancer diagnoses in the United States [2]. However, recent evidence has identified significant differences in the rate at which the NCDB captures incident cancer cases on the basis of several variables, including tumor, demographic, and geographic factors [4]. Because NCDB data are garnered from CoC-accredited programs, patients treated at non-CoC-accredited institutions are not reflected in this registry, and NCDB coverage rates are thus representative of access to CoC-accredited treatment facilities. Notably, CoC-accredited institutions are generally larger and have more cancer-related services available to patients compared to nonaccredited facilities [5]. Accordingly, differential NCDB case capture rates reflect potentially pertinent disparities in access to high-quality cancer treatment. 

In a recent analysis of NCDB case capture rates, Mallin et al. assessed data on more than 60 different primary tumors and found case capture rates to range from 51–93% in all patients on the basis of primary tumor location [4]. Substantial differences in the overall case capture rate were also observed for race (41–74%), sex (77% in females versus 68% in males), age (60–80%), and geographic region (56–85%). Among the top five most common cancer sites, the lowest coverage was found for prostate cancer (58%), and primary cancers for other genital organs were likewise below the average case coverage rate for all cancer sites combined. However, case rates for genitourinary cancers or other individual cancer sites were not ascertained for individual patient demographic subgroups. Notably, under-represented minorities are well known to face disparities in both access to urologic care and oncologic outcomes [6]. In view of the substandard NCDB coverage rate for prostate cancer and other genitourinary malignancies, as well as the presence of well-described racial disparities in urologic care, it stands to reason that access to CoC-accredited programs as a function of race merits further investigation. Accordingly, we aimed to characterize NCDB coverage rates for patients with genitourinary cancers across different demographic subpopulations.

## 2. Materials and Methods

As described above, the NCDB captures nearly 70% of all new cancer diagnoses in the United States, using data ascertained from more than 1500 CoC-accredited cancer treatment centers across the country [2]. In accordance with existing literature [4], the NCDB case capture rate was determined by comparing the incidence of genitourinary cancers in the NCDB to contemporary United States Cancer Statistics (USCS) data [7]. The USCS is an official government source for cancer statistics, comprised of combined data from the National Program of Cancer Registries (NPCR) and the Surveillance, Epidemiology, and End Results Program (SEER) [8]. Funded by the CDC, The NPCR is a population-based surveillance system of cancer registries funded by the Centers for Disease Control and Prevention which collects cancer data on the entire United States population using strict data quality standards [9]. SEER is a population–based surveillance system funded by the National Cancer Institute which collects data on cancer for 28% of the U.S population [10]. Thus, using pooled data from the NPCR and SEER, the USCS is widely regarded as the most complete and comprehensive cancer statistics dataset in the United States. Owing to the publicly available, deidentified nature of these datasets, institutional review board approval was not required.

We queried the NCDB and USCS to identify patients diagnosed with primary genitourinary malignancies from 2004–2015. Specific diagnoses considered included prostate, penile, testicular, bladder, and kidney cancer. As race categories are narrower/more specific in the NCDB compared to USCS data, NCDB race categories were recoded in order to standardize the categorization of race (Table 1), resulting in four major categories: White, Black, Asian/Pacific Islander (API), and American Indian/Alaskan Natives (AI/AN)). (In the USCS, “Hispanic” is coded as ethnicity, rather than race, precluding a direct comparison of coverage rates between self-identified Hispanic subjects and other groups.) Subjects were stratified based on race, and those listed as “unknown”/“other” were excluded. The coverage rates for non-sex-specific genitourinary cancers were also determined separately for males and females. 

A chi-squared test was used to compare the number of captured subjects of each race for each genitourinary malignancy. When intergroup differences were found to be significant, standard 2 × 2 chi-squared tests were used to determine the partial order between sample pairs. A *p*-value < 0.05 was deemed statistically significant and a Bonferroni correction was applied for multiple comparisons. Analyses were performed using SPSS Software Version 26.0 (IBM Corp., Armonk, NY, USA).

## 3. Results

Analysis of USCS data identified a total of 2,393,370 eligible subjects with prostate cancer, 14,822 with penile cancer, 96,764 with testicular cancer, 819,681 with bladder cancer, and 629,073 with kidney cancer (Table 2). Analysis of concurrent NCDB data revealed 1,346,813 eligible patients with prostate cancer (overall capture rate 56.3%), 12,202 with penile cancer (82.3%), 64,115 with testicular cancer (66.3%), 517,144 with bladder cancer (63.1%), and 456,508 with renal cancer (72.6%). In achieving the final study sample, fewer than 3% of subjects across all diagnoses were excluded from either database due to missing race data.

Upon subgroup analysis of NCDB capture rate by race, the observed distribution differed significantly from the expected distribution (*p* < 0.001) for all genitourinary malignancies (Table 3). AI/AN subjects demonstrated the lowest capture rate for all genitourinary cancers combined (32.6%) as well as across all individual malignancies—significantly below that of all other groups in all pairwise comparisons (*p* < 0.001). API subjects exhibited the second-to-lowest capture rates overall (53.7%) and across all malignancies—significantly higher than AI/AN subjects but also significantly lower than either White or Black subjects across all pairwise comparisons (*p* < 0.001).

Between White and Black subjects, White subjects had a significantly higher overall capture rate for genitourinary cancer (61.4% vs. 57.2%, *p* < 0.001). The prostate cancer capture rate was significantly higher in White subjects (57.1% vs. 53.2%, *p* < 0.001). No significant differences were observed for penile cancer (*p* = 0.515) or testicular cancer (*p* = 0.102). The kidney cancer capture rate was significantly higher in White subjects (73.3% vs. 71.7%, *p* < 0.001), with a significant difference observed in White males compared to Black males (72.9% vs. 70.1%, *p* < 0.001), but no significant difference observed in White females compared to Black females (*p* = 0.600). Conversely, the bladder cancer capture rate was significantly lower in White subjects (63.3% vs. 64.3%, *p* < 0.001), with a significantly lower rate in White females compared to Black females (65.5% vs. 69.0%, *p* < 0.001), but no significant difference was observed between White males and Black males (*p* = 0.069).

## 4. Discussion

The present study marks the first, to our knowledge, to ascertain NCDB capture rate for genitourinary cancers specifically as a function of race. Across all malignancies, AI/AN subjects demonstrated the lowest capture rates, and API subjects exhibited the second-lowest capture rates. Further, our analysis revealed several significant differences in NCDB genitourinary cancer capture rate between White and Black Americans, including a significantly higher rate for genitourinary malignancies overall in White patients, a significantly higher capture rate of prostate cancer and kidney cancer in White males, and a significantly higher capture rate of bladder cancer in Black females. 

The present study findings are consistent with existing evidence of substantial variation in the NCDB case capture rate on the basis of race and other demographic factors [4,11]. Interestingly, however, the present study results appear to diverge from prior findings with respect to the relative coverage rate in White and Black subjects. Namely, multiple prior analyses, which compared the pooled NCDB case capture rate for all cancers rather than rates for specific malignancies individually, found overall cancer capture rates to be similar between White and Black subjects. For example, an analysis of 2004–2006 NCDB and USCS data from Lerro et al. reported a comparable NCDB cancer coverage rate for Black (67.4%) and White Americans (67.1%) [11]. A similar analysis of 2012–2014 NCDB data from Mallin et al. found a 73.5% coverage rate in Black Americans compared to a 72.6% coverage rate in White Americans [4]. Although Mallin et al. did not apply any statistical tests to compare coverage rate frequencies between groups, based on their absolute frequency data, coverage rates would appear to significantly favor Black (381,341/519,153) versus White (2,902,931/3,995,821) subjects (*p* < 0.001) if our study methods for comparing sample pairs were employed. In contrast, although we did find bladder cancer coverage to be significantly higher in Black versus White subjects, White subjects demonstrated a significantly greater coverage rate for both prostate cancer and kidney cancer, corresponding to a significantly greater overall genitourinary cancer coverage rate in White versus Black subjects (61.4% vs. 57.2%, *p* < 0.001). Whether differential NCDB coverage for White and Black patients is unique to genitourinary cancer or a consistent finding for cancers in other body systems merits further investigation.

Our novel finding of a significantly higher case coverage rate for White versus Black Americans with genitourinary cancers is noteworthy in view of the well-described inferior treatment and survival outcomes among Black patients with these malignancies [6,12,13,14,15]. Interestingly, evidence demonstrating the presence and impact of racial disparities in prostate cancer and kidney cancer is particularly robust [13,14,15,16,17,18,19,20,21,22], and it is these two malignancies which appear to be underlying the significantly higher overall genitourinary case capture rate in White versus Black subjects observed in the present analysis. Although disparities in urologic oncology are multifactorial and complex, differential access to cancer care undoubtedly plays a role in this phenomenon, and research derived from an equal-access healthcare system suggests that improved access to care may be key in mitigating racial disparities in genitourinary cancer outcomes [23]. As CoC-accredited institutions generally have more cancer-related resources available to patients compared to nonaccredited facilities [5], and the NCDB coverage rate closely aligns with access to a CoC-accredited treatment center (with the reporting of all eligible cancer cases being a requirement to maintain CoC accreditation) [24], the present study observations of differential coverage rates by race across all genitourinary cancers constitute a novel avenue for health equity research. 

Importantly, while the NCDB is indeed a comprehensive repository of oncologic data, it is perhaps most noteworthy as a powerful benchmark of hospital performance [2]. The quality-of-care reports provided to participating institutions are not intended to be punitive or a source of accountability but are rather meant to provide comprehensive information to hospitals with the aim of improving patient outcomes within the framework of CoC standards for high-quality cancer care [2]. It should also be noted, however, that there exist several well-described barriers to CoC participation, including an increasing number of required patient-centered services that may not be reimbursable, as well as the perception of a relatively burdensome accreditation process [25]. Correspondingly, more widespread participation in the CoC accreditation process (which, as the present analysis suggests, stands to have the greatest effect on genitourinary cancer care for racial minorities) is predicated on greater evidence of a positive return from a hospital’s involvement, coupled with a lower perceived burden of participation [26]. Recent studies have highlighted the value of CoC standards in the management of colon cancer, demonstrating that improved compliance translates into increased survival [27,28], but similar evidence is still needed to test this hypothesis in the context of other malignancies including genitourinary cancers. Evidence from European accreditation bodies suggests that a simplified set of criteria may provide sufficient information on hospital performance, which could feasibly mitigate the perceived burden of the accreditation process, but we are unaware of similar analyses related to cancer care in the United States [29,30]. CoC standards are updated periodically, and several key changes were made in the most recent (2020) version relative to the prior (2016) rendition [31], but it remains to be seen what effect these changes may have on the perceived positive return and burden from participation. The present study findings affirm that particular attention must be afforded to hospitals serving diverse patient populations in evaluating the effects of CoC standards on patient outcomes.

Consistent with existing literature, coverage rates for AI/AN patients were consistently the lowest across all genitourinary cancers in the present analysis, and coverage rates for API patients likewise fell below that of either White or Black patients for all genitourinary malignancies [4,11]. Although AI/AN patients remain highly underrepresented in the urologic oncology literature, available evidence suggests that Native Americans with prostate or kidney cancer have significantly poorer survival compared to their White contemporaries [32,33]. Interestingly, while API patients have been reported to experience higher mortality from testicular cancer [34], existing literature suggests that these patients have favorable outcomes for most genitourinary cancers compared to White patients, including a lower incidence and cancer-specific mortality from prostate cancer, kidney cancer, bladder cancer, and penile cancer [13,15,35,36]. However, for both AI/AN and API patients, under-representation in the NCDB may not only be reflective of differential access to care but also of direct relevance to current NCDB research seeking to address pressing knowledge gaps in the urology and oncology literature. For example, one recent NCDB analysis reported that certain histopathologic variants of kidney cancer occurred less commonly in AI/AN and API patients compared to White patients [37]. Although the authors acknowledged racial differences in NCDB coverage rate as a major limitation, aptly citing Mallin et al. and other relevant literature in their discussion section, our observed genitourinary cancer case capture rates (39% and 64% in AI/AN and API patients, respectively) fell even further below the case capture rate reported by Mallin et al. for these groups [4]. The substandard NCDB case capture rates for AI/AN and API patients, particularly in the context of genitourinary cancers, underscores the potential pitfalls of generalizing NCDB data to inform treatment decision-making for these underrepresented groups. 

The present study findings must be interpreted in view of the limitations of a retrospective registry analysis [24]. As with other registry analyses, the present retrospective observational study results assume consistent and accurate variable coding. The potential for misclassification of race, particularly among individuals who identify as AI/AN, is another inherent limitation of cancer registry data [38]. Although the quality standards on which CoC accreditation is predicated are widely considered to have a positive effect on patient outcomes and survival [39], other facility factors, such as operative volume, may also directly impact patient outcomes [40]. Further, even among patients treated at CoC-accredited facilities, equal access may not always translate to equal care, as some recent NCDB analyses have challenged the potential role of race on prostate cancer outcomes, while others have identified Black race, female sex, and other patient demographic factors as predictors of less timely therapy and poorer survival in bladder cancer [41,42]. How patient race may intersect with geography, another known modifier of NCDB case coverage rate, also requires further study.

## 5. Conclusions

The present study findings of disparate representation of AI/AN and API genitourinary cancer patients in the NCDB—falling below that observed in prior studies on overall cancer capture rates—underscore the fact that NCDB results specifically derived from these groups must be interpreted judiciously in treatment decision-making. The observed significant difference in prostate, kidney, and bladder cancer coverage rates between White and Black subjects—a finding not previously reported in prior studies on overall NCDB cancer capture rates—may also provide a valuable foundation for future research on access to genitourinary cancer care for Black Americans. 

## Figures and Tables

**Table 1:** Comparison of race classification between United States Cancer Statistics data and the National Cancer Database.

USCS Category	Associated NCDB Categories
- American Indian/Alaska Native	- American Indian, Aleutian, or Eskimo
- Asian or Pacific Islander	- Chinese - Japanese - Filipino - Hawaiian - Korean - Vietnamese - Laotian - Hmong - Kampuchean - Thai - Asian Indian or Pakistani, NOS - Asian Indian - Pakistani - Micronesian, NOS - Chamorran - Guamanian, NOS - Polynesian, NOS - Tahitian - Samoan - Tongan - Melanesian, NOS - Fiji Islander - New Guinean - Other Asian, including Asian, NOS and Oriental, NOS - Pacific Islander, NOS

**Note:** As race categories are narrower/more specific in the NCDB compared to USCS data, NCDB race categories were recoded in order to standardize the categorization of race, resulting in four major categories: White, Black, Asian/Pacific Islander (API), and American Indian/Alaskan Natives (AI/AN)). Abbreviations: NCDB, National Cancer Database; NOS, Not Otherwise Specified; USCS, United States Cancer Statistics.

**Table 2:** Capture rate of incident genitourinary cancer cases in the National Cancer Database.

Primary Tumor	Eligible Subjects—NCDB	Eligible Subjects—USCS	Total Capture Rate
Prostate Cancer	1,346,813	2,393,370	56.3%
Penile Cancer	12,202	14,822	82.3%
Testicular Cancer	64,115	96,764	66.3%
Kidney Cancer	456,508	629,073	72.6%
Males	282,614	392,175	72.1%
Females	173,894	236,898	73.4%
Bladder Cancer	517,144	819,681	63.1%
Males	386,443	620,008	62.3%
Females	130,701	199,673	65.5%

**Note:** Total capture rate computed as cancer incidence in the NCDB divided by cancer incidence according to USCS data. Abbreviations—NCDB, National Cancer Database; USCS, United States Cancer Statistics.

**Table 3:** Incident genitourinary cancer capture rate stratified by race and sex.

Primary Cancer	Capture Rate				*p*-Value—All Groups	*p*-Value—White vs. Black
	White Subjects	Black Subjects	API	AI/AN		
All GU Cancers	61.4% (2,072,409/3,373,359)	57.2% (276,444/483,662)	53.7% (41,768/77,764)	32.6% (6161/18,925)	<0.001 *	<0.001 *
Prostate	57.1% (1,126,439/1,971,925)	53.2% (192,786/362,429)	50.2% (24,651/49,077)	29.6% (2937/9939)	<0.001 *	<0.001 *
Penile	82.8% (10,607/12,813)	83.4% (1305/1564)	71.6% (239/334)	46.0% (51/111)	<0.001 *	0.515
Testicular	66.8% (60,556/90,664)	65.4% (2139/3269)	56.1% (1100/1961)	35.6% (310/870)	<0.001 *	0.102
Kidney Cancer	73.3% (394,074/537,996)	71.7% (52,015/72,574)	63.5% (8273/13,028)	39.2% (2146/5475)	<0.001 *	<0.001 *
Males	72.9% (245,577/337,023)	70.1% (30,523/43,544)	63.4% (5282/8330)	37.6% (1232/3278)	<0.001 *	<0.001 *
Females	73.9% (148,497/200,973)	74.0% (21,492/29,030)	63.7% (2991/4698)	41.6% (914/1283)	<0.001 *	0.600
Bladder Cancer	63.3% (480,723/759,961)	64.3% (28,199/43,826)	56.2% (7505/13,364)	28.3% (717/2530)	<0.001 *	<0.001 *
Males	62.6% (362,138/578,811)	62.0% (18,131/29,226)	56.1% (5644/10,064)	27.8% (530/1907)	<0.001 *	0.069
Females	65.5% (118,585/181,150)	69.0% (10,068/14,600)	56.4% (1861/3300)	30.0% (187/623)	<0.001 *	<0.001 *

**Note:** Capture rate computed as cancer incidence in the NCDB divided by cancer incidence according to USCS data. * Denotes statistical significance (*p* < 0.001). On pairwise comparison, the capture rate among AI/AN subjects was significantly lower compared to all other groups for all genitourinary cancers combined as well as across all individual malignancies (*p* < 0.001 for all). Pairwise comparison also demonstrated the capture rate among API subjects to be significantly higher than among AI/AN subjects and significantly lower than among White and Black subjects for all genitourinary cancers combined as well as across all individual malignancies (*p* < 0.001 for all). Abbreviations—API, Asian/Pacific Islander; AI/AN, American Indian/Alaskan Natives; GU, Genitourinary.

## Data Availability

The present dataset was derived from a publicly available database.
